# Associations between serum estradiol and estrone and Alzheimer's disease biomarkers: An analysis in female participants from the European Prevention of Alzheimer's Dementia Longitudinal Cohort Study (EPAD LCS)

**DOI:** 10.1002/alz.71826

**Published:** 2026-09-13

**Authors:** Juehyun Shin, Graciela Muniz‐Terrera, Craig W. Ritchie, Jean Manson, Susan Plachecki, Clemens Kirschbaum, Sarah Gregory

**Affiliations:** ^1^ Heritage College of Osteopathic Medicine Ohio University Athens Ohio USA; ^2^ The Alzheimer's Pathobiome Initiative (AlzPI) Wake Forest North Carolina USA; ^3^ Scottish Brain Sciences Edinburgh UK; ^4^ University of St Andrews St Andrews UK; ^5^ University of Edinburgh Edinburgh UK; ^6^ Dresden LABservice GmbH Dresden Germany

**Keywords:** Alzheimer's disease, amyloid, APOE, CSF biomarkers, estradiol, estrogen, estrone, tau, women's health

## Abstract

**INTRODUCTION:**

Postmenopausal estrogen decline may contribute to Alzheimer's disease (AD), but evidence linking circulating estrogens to cerebrospinal fluid (CSF) AD biomarkers remains limited.

**METHODS:**

We analyzed 854 female participants from the European Prevention of Alzheimer's Dementia Longitudinal Cohort Study with baseline serum estradiol and estrone measured by liquid chromatography–tandem mass spectrometry and repeated CSF amyloid‐beta 42 (Aβ_42_), phosphorylated tau 181 (pTau_181_), and total tau (tTau). Models used regression for cross‐sectional analyses and mixed‐effects models restricted to the 187 participants with follow‐up, adjusted for age, body mass index, waist circumference, and hormone replacement therapy (HRT) use. We also tested apolipoprotein E *ε4* (*APOEε4*) and amyloid moderation and conducted sensitivity analyses excluding current HRT users.

**RESULTS:**

Neither hormone was associated with Aβ_42_. Higher estradiol was associated with lower baseline tau, especially among *APOEε4* carriers, but not with tau change. In exploratory longitudinal analyses, estrone showed a modest association with slower pTau_181_ increase.

**DISCUSSION:**

Findings suggest more consistent cross‐sectional associations of estradiol with tau than with Aβ_42_; longitudinal findings were limited by attrition and should be interpreted as exploratory.

## Highlights

- Liquid chromatography–tandem mass spectrometry (LC‐MS/MS) quantified low‐concentration serum estrogens in European Prevention of Alzheimer's Dementia Longitudinal Cohort (EPAD LCS) Study
- Higher estradiol was associated with lower cerebrospinal fluid (CSF) tau at baseline
- Estradiol‐tau associations were stronger in apolipoprotein E ε4 (*APOEε4)* carriers at baseline.
- Neither estrogen was associated with CSF amyloid‐beta 42 (Aβ_42_)

## 1 BACKGROUND

Globally, dementia prevalence is consistently higher in females than males, beginning in late midlife with the gap widening across older age groups.^1^ Consistent with this epidemiologic pattern, females comprise a larger share of individuals living with Alzheimer's disease (AD) dementia, and autopsy‐based studies suggest females can show higher overall AD neuropathology, particularly greater tau tangle density.^2^ Analyses from the Religious Orders Study and the Memory and Aging Project demonstrated that female participants had greater tau tangle burden than males across multiple brain regions, even after propensity score matching on demographics, apolipoprotein E *ε4* (*APOEε4*) allele count, and other key factors.^3^

Beyond longevity, biological sex differences, particularly sex hormones, have emerged as critical factors that may explain these disparities in AD pathogenesis and risk.^4^, ^5^, ^6^, ^7^ Preclinical work suggests that estrogens are neuroactive and may exert neuroprotective effects relevant to AD, including reducing amyloid‐beta (Aβ) ‐related toxicity and modulating tau hyperphosphorylation.^8^, ^9^, ^10^, ^11^ The menopausal transition is characterized by a rapid decline in endogenous estrogens, which has been hypothesized to increase vulnerability to AD‐relevant pathophysiology in females.^7^, ^9^, ^10^, ^11^ Observational work further suggests that shorter lifetime endogenous estrogen exposure and earlier menopause may be associated with elevated risk of cognitive impairment and dementia outcomes.^9^, ^12^ Consistent with a menopause‐timing framework that emphasizes downstream tau vulnerability, autopsy evidence indicates that earlier menopause can amplify the association between reduced synaptic integrity and tau tangle burden.^12^

While preclinical evidence supports estrogen's neuroprotective role, translating these findings to human populations has proven challenging. Human studies examining estrogen‐AD relationships (including biomarker and cognitive endpoints) and hormone replacement therapy (HRT) have yielded mixed findings. This heterogeneity likely reflects differences in the timing of initiation relative to menopause (including the proposed “critical window”), formulation (e.g., estrogen‐only vs. estrogen‐progestogen therapy), and baseline characteristics such as genotype and cardiovascular health.^7^, ^9^, ^10^, ^11^, ^13^, ^14^ A major limitation is reliance on indirect proxies of estrogen exposure (e.g., reproductive history, menopausal status, or HRT use) rather than direct measurement of circulating hormone levels and receptor‐mediated signaling activity.^8^, ^9^

The *APOEε4* allele is the strongest common genetic risk factor for late‐onset AD, and its effects appear sex‐dependent, with female *APOEε4* carriers showing higher AD risk, earlier onset, and faster decline than male carriers.^3^, ^15^, ^16^, ^17^ *APOEε4* has also been linked to greater tau burden, tau‐related neurodegeneration, and a more proinflammatory microglial profile beyond its influence on amyloid,^18^, ^19^, ^20^, ^21^ and estrogen‐related sterol pathways may differ by *APOEε4* status,^17^ suggesting that *APOEε4* could shape how estrogens relate to tau pathology. Further, literature shows greater amyloid burden drives downstream tau accumulation, a process that may be more pronounced in females.^22^, ^23^, ^24^, ^25^, ^26^

Studies examining circulating estrogens in relation to cerebrospinal fluid (CSF) AD biomarker trajectories in this population are lacking. The present study addresses this gap by examining associations between serum estrogen levels (estradiol and estrone) and CSF biomarkers of AD pathology in females which are early‐changing biomarkers that reflect AD‐specific pathophysiology emerging early along the disease continuum.^27^ We hypothesized that, in females, higher serum estradiol and estrone levels would be associated with slower longitudinal increases in CSF AD biomarkers, amyloid‐beta 42 (Aβ_42_), phosphorylated tau 181 (pTau_181_), and total tau (tTau), and that these associations would differ by *APOEε4* carrier status and amyloid positivity.

**RESEARCH IN CONTEXT**

1. **Systematic review**: We reviewed PubMed and reference lists for studies of estrogens, hormone therapy, apolipoprotein E *ε4* (*APOEε4)*, and cerebrospinal fluid (CSF) biomarkers of Alzheimer's disease (AD)in women and aging populations.
2. **Interpretation**: Prior work suggests estrogen‐related biology may influence AD pathology, but human biomarker evidence remains limited. In European Prevention of Alzheimer's Dementia Longitudinal Cohort (EPAD LCS) female participants, liquid chromatography–tandem mass spectrometry (LC‐MS/MS) ‐measured serum estradiol was associated with lower baseline CSF tau, particularly among *APOEε4* carriers, whereas neither estradiol nor estrone was associated with CSF amyloid‐beta 42 (Aβ_42_). These cross‐sectional findings were more consistent than the longitudinal findings, which were limited by substantial attrition and should be interpreted as exploratory.
3. **Future directions**: Studies should test whether repeated serum and CSF estrogens, menopausal timing, HRT characteristics, and metabolic markers explain tau‐related vulnerability.

## 2 METHODS

### 2.1 Participants

**FIGURE 1:**
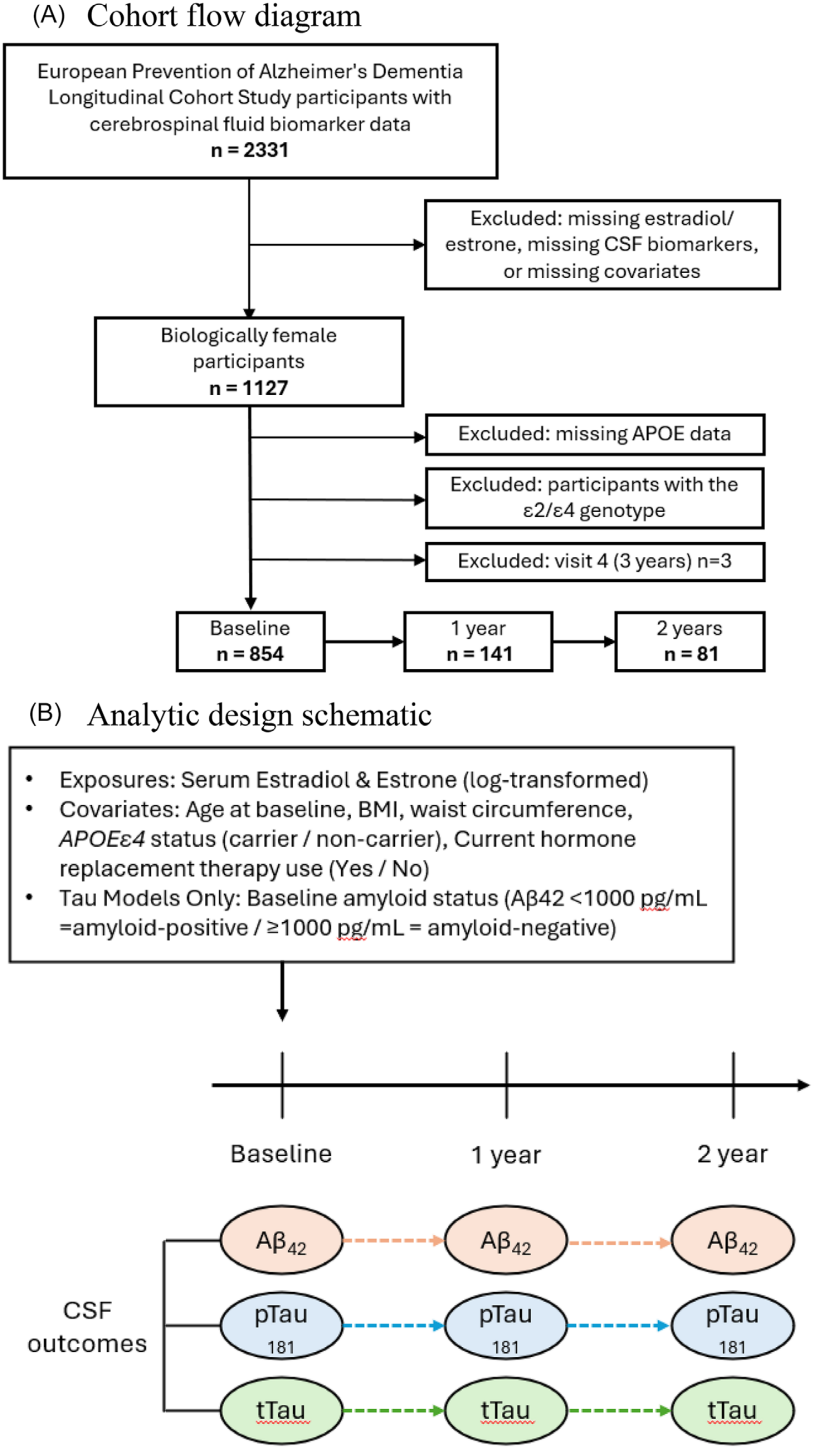
Participant flow diagram and analytic design schematic.

This study utilized data and biological samples from biologically female participants in the European Prevention of Alzheimer's Dementia Longitudinal Cohort Study (EPAD LCS). The EPAD LCS enrolled individuals aged 50+ years representing a spectrum of risk for AD dementia, spanning from healthy volunteers to those with mild cognitive impairment.^28^, ^29^, ^30^ Participants were excluded if they had missing data on demographic characteristics, *APOEε4* carrier status, estrogen measures (estradiol or estrone), or CSF biomarkers (Aβ_42_, pTau_181_, or tTau). The participant flow diagram (Figure 1A) summarizes how the analytic sample was derived from the EPAD LCS cohort. All participants provided informed consent, and local ethical approval was obtained from ethics committees at each research site. All procedures were conducted in accordance with the Helsinki Declaration.

### 2.2 Estradiol and estrone measurement

Blood samples collected as part of the EPAD LCS were analyzed for estradiol and estrone (measured specifically for the present study) at Dresden LABservice GmbH (Dresden, Germany) using liquid chromatography tandem mass spectrometry (LC‐MS/MS); details of the online solid‐phase extraction–high‐performance liquid chromatography (SPE HPLC) method have been reported previously.^31^ In contrast to the instrumentation and mobile phases described, the analysis was performed on a SCIEX 6500+ triple quad mass spectrometer.

The present method used 100% methanol as mobile phase A, 0.2 mM ammonium fluoride in water as mobile phase B, C‐A was water (first elution step and re‐equilibration step), and C‐B was 100% LC‐MS grade methanol (washing columns and needle). The flow rate was maintained at 3 mL/minute, with a column temperature of 40°C, an injection volume of 50 µL, with a total run time of 9 minutes. The LC eluent was introduced to the MS through the turbo‐ion‐spray probe in negative ionization mode for estrogens, with multiple reaction monitoring (MRM) used. The following ion source parameters were used: curtain gas (CUR) set to 35, collision gas (CAD) was set to 7, ion spray voltage (IS) was ‐4500 V, ion source temperature (TEM) was 350°C, and ion source gas 1 and ion source gas 2 were both set to 65.

A quantifier and a qualifier ion were detected for each analyte in each sample. Established calibration curves were used for quality control, alongside quality control samples included in the runs with known concentrations of analytes of interest to check for accuracy of detection. For quality control (QC), in‐house serum QC samples were used due to the lack of commercially available QC material for estrogen. In‐house QC samples were prepared from a single production batch, stored at ‐80°C, and thawed immediately before use. Because synthetic serum was not available, commercially available charcoal‐stripped fetal bovine serum was used as the serum matrix. System QC samples were used to calculate assay accuracy, which ranged from 100% to 102%. The limit of detection (LOD) and limit of quantification (LOQ) were determined using fetal bovine serum as follows: estradiol LOD 0.67 pg/ml and LOQ 2.04 pg/ml; estrone LOD 0.99 pg/ml and LOQ 3.00 pg/ml; estriol LOD 0.57 pg/ml and LOQ 1.74 pg/ml. Inter‐assay and intra‐assay precision were summarized using coefficient of variation (CV) values: estradiol inter‐assay %CV 5.4 and 3.4, intra‐assay %CV 2.5 and 2.7, accuracy 102.6%; estrone inter‐assay %CV 3.8 and 3.7, intra‐assay %CV 2.3 and 2.1, accuracy 100.7%; estriol inter‐assay %CV 5.3 and 3.7, intra‐assay %CV 1.8 and 2.0, accuracy 100.8%. Circulating estrogen levels (estradiol and estrone) were measured as continuous variables. Estriol was also measured, but because it was below the assay LOD (LOD = 0.57 pg/ml) in most participants and quantifiable in only *n* = 5, no statistical analysis was carried out using this data.

### 2.3 CSF biomarker assessment

CSF samples were analyzed at the Clinical Neurochemistry Laboratory, University of Gothenburg, Sweden, using the Roche Diagnostics Elecsys platform for Alzheimer's disease biomarkers, including Aβ_42_, pTau_181_, and tTau. The established cutoffs for the EPAD LCS are defined as: Aβ_42_ < 1000 pg/ml (amyloid‐positive).^32^ For data processing, values below the reporting limits were set to the limit (pTau_181_ = 8 pg/ml, tTau = 80 pg/ml, Aβ_42_ = 200 pg/ml), and Aβ_42_ values > 1700 pg/ml were handled using recalculated results.

### 2.4 *APOE* genotyping

*APOEε4* data were categorized into two groups: a non‐carrier group (including *ε2/ε2*, *ε2/ε3*, and *ε3/ε3* genotypes) and a carrier group (including *ε3/ε4* and *ε4/ε4* genotypes). Participants with the *ε2/ε4* genotype were excluded from the analysis as this genotype includes both protective and risk alleles.^33^

### 2.5 Hormone replacement therapy status

HRT use was identified from recorded medication data at baseline. Participants were classified as current HRT users based on recorded use of estrogen‐containing preparation at baseline. Because the number of current users was small, HRT was analyzed as a binary variable (current use: yes/no), and formulation‐, dose‐, route‐, and duration‐specific effects were not evaluated.

### 2.6 Statistical analysis

Because baseline estradiol and estrone distributions were right‐skewed, hormone concentrations were natural log‐transformed (ln‐transformed) for all analyses to improve distributional symmetry and model linearity; distributional diagnostics before and after transformation are presented in the Supplement (Figure S1 and Table S1 in Supplement). Cross‐sectional associations between baseline (Visit 1) serum estrogens and CSF biomarkers were examined using linear regression in the full analytic sample. Longitudinal associations were examined using linear mixed‐effects models restricted to participants with at least one follow‐up visit, to improve interpretability of slope estimates given substantial attrition. Age at visit was used as the time metric in the longitudinal models and mean‐centered by subtracting the mean age across observations included in the follow‐up‐restricted longitudinal analytic sample. Thus, model intercepts correspond to expected biomarker levels at the centered mean age among participants with follow‐up, rather than to baseline biomarker levels. Primary longitudinal models excluded Visit 4 (Year 3) because only three participants contributed data at this visit. Mixed‐effects models included random intercepts and random slopes for centered age at visit, and time interaction terms were included for all covariates. Models adjusted for baseline age, *APOEε4* carrier status, body mass index (BMI), waist circumference, and current HRT use, and *APOEε4* carrier moderation was evaluated; tau models additionally included baseline amyloid‐positive status as covariate and tested amyloid moderation. Estradiol and estrone were analyzed in separate single‐hormone models, and each CSF biomarker was modeled separately; because the two estrogens are strongly correlated, entering them simultaneously would introduce multicollinearity. The *APOEε4* × hormone and amyloid‐positive status × hormone interaction terms were evaluated within these same separate models. Figure 1B provides a schematic overview of the analytic design, including timing of baseline hormone measurement and repeated CSF biomarker assessments, and the covariates included in both the cross‐sectional and longitudinal models. Given the focused, hypothesis‐driven analyses, results are presented using nominal p‐values with 95% CIs and were not corrected for multiple comparisons across the primary cross‐sectional and longitudinal models; accordingly, all findings should be interpreted as exploratory and hypothesis‐generating rather than confirmatory. As a sensitivity analysis addressing potential confounding by exogenous hormone use, we repeated the primary models after excluding participants who reported current HRT use at baseline. Analyses were conducted using Stata (version 19).

## 3 RESULTS

After exclusion of 21 participants with the ε2/ε4 genotype, the analytic sample comprised 854 participants. At baseline, mean age was 65.18 years (SD 7.21). Mean ln‐transformed estradiol and estrone concentrations were 2.41 (SD 0.99) and 4.86 (SD 0.48), respectively (Table 1a), corresponding to geometric means of approximately 11.1 pg/ml for estradiol and 129.0 pg/ml for estrone. Although absolute concentrations were low, both analytes showed substantial between‐participant variability. Back‐transformed concentrations spanned approximately 1.5‐81 pg/ml (estradiol) and 49‐338 pg/ml (estrone) within ± 2 SD of the geometric mean, an approximately 50‐fold and seven‐fold spread, respectively. Mean CSF Aβ_42_, Tau_181_, and tTau concentrations were 1430.89 pg/ml (SD = 749.04), 19.26 pg/ml (SD = 10.20), and 222.86 pg/ml (SD = 96.56), respectively. At baseline, 31.85% of participants were amyloid‐positive, 37.59% were *APOEε4* carriers, and 7.38% reported current HRT use. Sample size declined across follow‐up visits to 141 at Time 2 and 81 at Time3. Baseline characteristics were compared between participants with follow‐up data (*n* = 187) and those without follow‐up data (*n* = 667) (Table 1b). Participants with follow‐up had higher baseline ln‐transformed estradiol and estrone concentrations than those without follow‐up (both *p* < 0.001). Given these differences and the need for repeated observations to estimate within‐person biomarker change, longitudinal analyses were restricted to participants with at least one follow‐up visit.

**TABLE 1:** Sample characteristics of the analytic cohort.

a. Sample characteristics of each visit				
		Time1 (*n* = 854)	Time2 (*n* = 141)	Time3 (*n* = 81)
Variable	Category	M (SD), Min‐Max or N (%)	M (SD), Min‐Max or N (%)	M (SD), Min‐Max or N (%)
Age (years)		65.18 (7.21), 50.08‐85.67	67.14 (6.30), 52.08‐81.01	66.26 (5.82), 53.95‐80.61
Education (years)		14.15 (3.61), 5‐32	13.87 (3.93), 7‐25	14.11 (3.27), 8‐21
Body mass index		25.56 (4.64), 15.65‐52.03	25.79 (4.35), 17.63‐40.55	25.46 (4.62), 17.63‐39.38
Waist circumference (cm)		85.69 (13.49), 28‐178	87.18 (11.30), 63‐125	85.76 (11.68), 63‐115
Estradiol (ln‐transformed, pg/ml)		2.41 (0.99), ‐1.54‐5.44	2.81 (0.64), ‐0.31‐5.08	3.05 (0.46), 2.19‐5.08
Estrone (ln‐transformed, pg/ml)		4.86 (0.48), 3.70‐8.40	4.96 (0.50), 4.14‐8.10	5.02 (0.59), 4.37‐8.10
Aβ_42_ (pg/ml)		1430.89 (749.04), 200.00‐6828.00	1365.23 (748.78), 252.30‐4228.00	1515.94 (658.81), 341.10‐3110.00
pTau_181_ (pg/ml)		19.26 (10.20), 8.00‐97.82	20.14 (12.28), 8.00‐118.60	20.44 (11.78), 8.00‐83.34
tTau (pg/ml)		222.86 (96.56), 80.00‐918.10	229.28 (111.35), 80.00‐985.10	232.26 (111.37), 92.13‐832.80
Aβ_42_ status	Amyloid‐positive	272 (31.85%)	55 (39.01%)	22 (27.16%)
	Amyloid‐negative	582 (68.15%)	86 (60.99%)	59 (72.84%)
Ethnicity	Caucasian/White	634 (74.24%)	124 (87.94%)	66 (81.48%)
	Hispanic/ Latino	10 (1.17%)	0 (0%)	0 (0%)
	Black/Asian/Other	7 (0.82%)	0 (0%)	0 (0%)
	Unavailable	203 (23.77%)	17 (12.06%)	15 (18.52%)
*APOEε4* status	ε4 non‐carrier	533 (62.41%)	84 (59.57%)	41 (50.62%)
	ε4 carrier	321 (37.59%)	57 (40.43%)	40 (49.38%)
HRT current use	Yes	63 (7.38%)	13 (9.22%)	9 (11.11%)
	No	791 (92.62%)	128 (90.78%)	72 (88.89%)

b. Baseline characteristics of participants with and without follow‐up data				
Variable	Category	With follow‐up (*n* = 187) M (SD) or N (%)	Without follow‐up(*n* = 667) M (SD) or N (%)	*p‐Value* (t‐test/χ2 test)
Age (years)		65.63 (6.27)	65.05 (7.45)	0.33
Education (years)		13.75 (3.76)	14.25 (3.56)	0.09
Body mass index		25.81 (4.44)	25.49 (4.70)	0.41
Waist circumference (cm)		87.11 (11.45)	85.29 (13.99)	0.10
Estradiol (ln‐transformed, pg/ml)		2.87 (0.59)	2.28 (1.04)	<0.001^***^
Estrone (ln‐transformed, pg/ml)		4.96 (0.46)	4.83 (0.48)	<0.001^***^
Aβ_42_ (pg/ml)		1337.15 (643.12)	1457.17 (774.54)	0.05
pTau_181_ (pg/ml)		19.49 (11.58)	19.19 (9.79)	0.73
tTau (pg/ml)		224.23 (104.20)	222.47 (94.39)	0.83
Aβ_42_ status	Amyloid‐positive	63 (33.69%)	209 (31.33%)	0.54
	Amyloid‐negative	124 (66.31%)	458 (68.67%)	
Ethnicity	Caucasian/White	161 (86.10%)	473 (70.91%)	<0.001^***^
	Hispanic/Latino	0 (0%)	10 (1.50%)	
	Black/Asian/Other	0 (0%)	7 (1.05%)	
	Unavailable	26 (13.90%)	177 (26.54%)	
*APOEε4* status	ε4 non‐carrier	111 (59.36%)	422 (63.27%)	0.33
	ε4 carrier	76 (40.64%)	245 (36.73%)	
HRT current use	Yes	15 (8.02%)	48 (7.20%)	0.70
	No	172 (91.98%)	619 (92.80%)	

Abbreviations: Aβ_42_, amyloid‐beta 42; *APOEε4*, apolipoprotein E *ε4*; HRT, hormone replacement therapy; LOD, limit of detection; pTau_181_, phosphorylated tau 181; SD, standard deviation; tTau, total tau.

*Note*: Estriol was below the assay LOD (0.57 pg/ml) in most participants (quantifiable in *n* = 5) and was not analyzed.

* *p *< 0.05.

** *p *< 0.01.

*** *p *< 0.001.

### 3.1 Estradiol / Estrone and Aβ_42_

In baseline cross‐sectional analyses at Visit 1 (Table 2a; covariate estimates in Table S2a of the Supplement), ln‐transformed estradiol and estrone were not associated with baseline CSF Aβ_42_. There was no evidence that *APOEε4* carrier status modified baseline hormone‐Aβ_42_ associations. In longitudinal mixed‐effects models restricted to participants with follow‐up (Table 2b; covariate estimates in Table S2b of the Supplement), neither baseline estradiol nor baseline estrone was associated with change in Aβ_42_ over time, and *APOEε4* did not modify hormone‐Aβ_42_ slope associations.

**TABLE 2:** Baseline cross‐sectional and longitudinal associations of serum estradiol and estrone with CSF Aβ_42_.

Outcome	Primary exposures	*B* (95% CI)	SE	*p*‐value
**a. Baseline cross‐sectional (Visit 1; *n* = 854)**				
Aβ_42_ (baseline)	ln (estradiol)	−33.60 (−86.37, 19.16)	26.88	0.212
	ln (estradiol) × *APOEε4*	−0.61 (−101.67, 100.44)	51.49	0.991
	ln (estrone)	28.35 (−74.08, 130.78)	52.19	0.587
	ln (estrone) × *APOEε4*	1.79 (−187.91, 191.49)	96.65	0.985
**b. Longitudinal change (≥ 1 follow‐up; *n* = 187)**				
Aβ_42_	ln (estradiol) × time	5.17 (−16.21, 26.55)	10.91	0.636
	ln (estradiol) × *APOEε4* × time	−3.92 (−40.69, 32.85)	18.76	0.834
	ln (estrone) × time	6.74 (−21.72, 35.20)	14.52	0.643
	ln (estrone) × *APOEε4* × time	−4.23 (−56.50, 48.03)	26.67	0.874

*Notes*: (a) Separate single‐hormone models; each biomarker and each interaction term modeled separately. Linear regression models adjusted for baseline age (years), BMI, waist circumference, current hormone replacement therapy use, and *APOEε4* carrier status; interaction rows represent the additional difference in the hormone association for APOE ε4 carriers versus non‐carriers, respectively. Robust (Huber–White) standard errors were used. *B* indicates the unstandardized regression coefficient. ln () indicates natural log‐transformed values.

(b) Separate single‐hormone models; each biomarker and each interaction term modeled separately. Longitudinal mixed‐effects models were restricted to participants with at least one follow‐up visit and excluded Visit 4 (Year 3; *n* = 3). Age at visit was used as the time metric and mean‐centered; therefore, time terms represent associations with change in Aβ_42_ over follow‐up. Models were adjusted for baseline age, BMI, waist circumference, current hormone replacement therapy use, and *APOEε4* carrier status; interaction terms represent differences in hormone–slope associations by *APOEε4* carrier status. *B* indicates the unstandardized regression coefficient. ln () indicates natural log‐transformed values.

Abbreviations: Aβ, amyloid‐beta; *APOEε4*, apolipoprotein E *ε4*; BMI, body mass index; CI, confidence interval; CSF, cerebrospinal fluid; SE, standard error.

### 3.2 Estradiol / Estrone and pTau_181_

**FIGURE 2:**
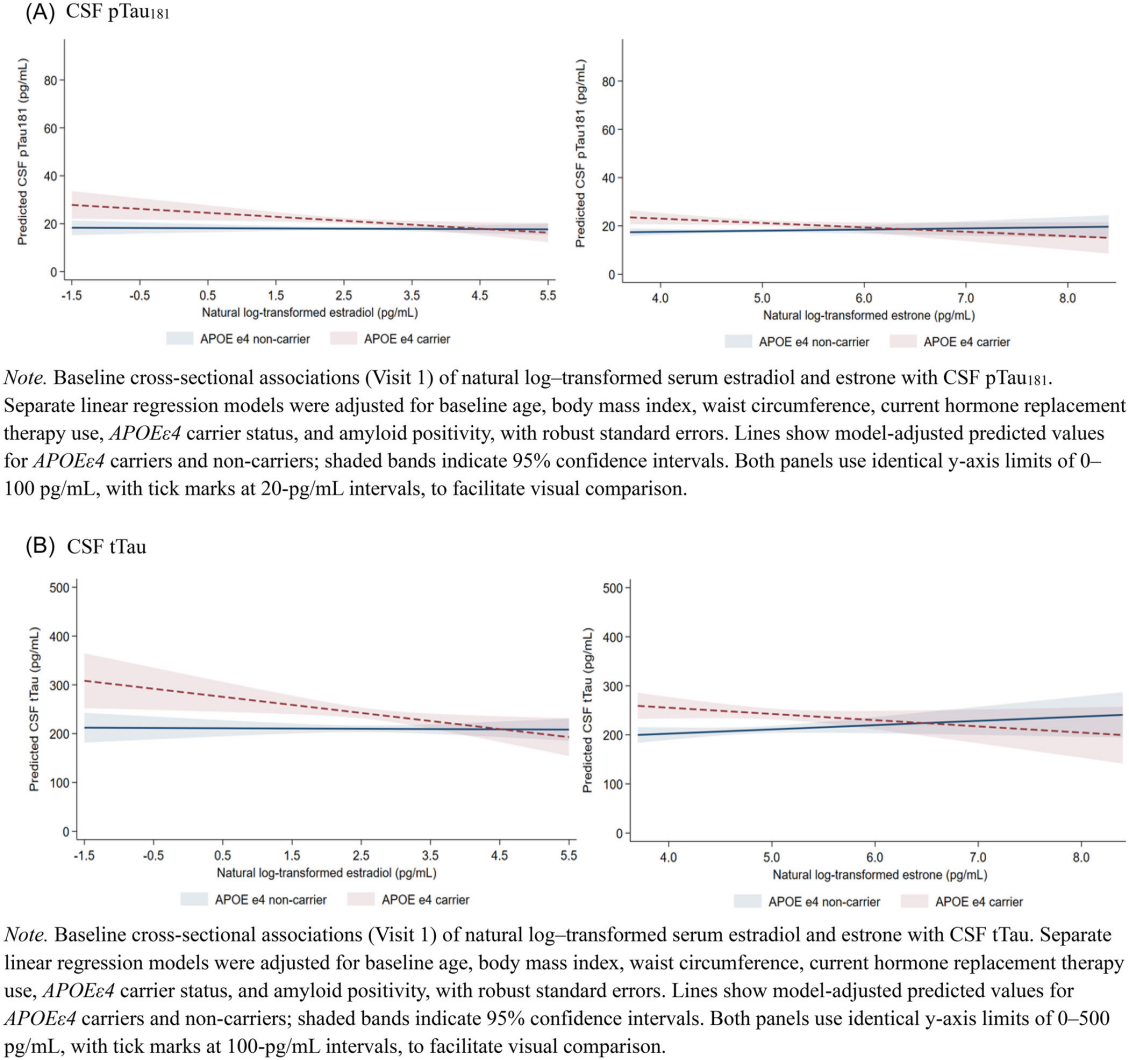
Associations between serum estrogens and CSF tau biomarkers by *APOEε4* carrier status at baseline. *APOEε4*, apolipoprotein E *ε4*; CSF, cerebrospinal fluid.

In baseline cross‐sectional analyses (Table 3a; covariate estimates in Table S3a), higher ln‐transformed estradiol was associated with lower baseline CSF pTau_181_ (*β* = −0.723, *p* = 0.049). Baseline estradiol–pTau181 associations were stronger among *APOEε4* carriers than non‐carriers (*β* = −1.557, *p* = 0.047; Figure 2A), with no evidence of modification by amyloid positivity. Baseline estrone was not associated with baseline pTau181, the estrone × *APOEε4* interaction was of borderline significance (*p *= 0.05; Figure 2A), whereas the estrone × amyloid positivity interaction was not statistically significant. In longitudinal models restricted to participants with follow‐up (Table 3b; covariate estimates in Table S3b), baseline estradiol was not associated with changes in pTau_181_ over time, and there was no evidence of effect modification by *APOEε4* or amyloid positivity status for estradiol slope terms. Baseline estrone was associated with a modest decrease in pTau_181_ over follow‐up (*β* = −0.54, *p* = 0.021), with no evidence of modification by *APOEε4* or amyloid positivity status.

**TABLE 3:** Baseline cross‐sectional and longitudinal associations of serum estradiol and estrone with CSF pTau_181_.

Outcome	Primary exposures	*B* (95% CI)	SE	*p*‐Value
**a. Baseline cross‐sectional (Visit 1; *n* = 854)**				
pTau_181_ (baseline)	ln (estradiol)	−0.72 (−1.445, −0.002)	0.37	0.049*
	ln (estradiol) × *APOEε4*	−1.56 (−3.096, −0.019)	0.78	0.047*
	ln (estradiol) × Aβ+	−0.88 (−2.50, 0.75)	0.83	0.289
	ln (estrone)	−0.49 (−1.629, 0.643)	0.58	0.394
	ln (estrone) × *APOEε4*	−2.29 (−4.566, −0.005)	1.16	0.05
	ln (estrone) × Aβ+	−1.87 (−5.21, 1.48)	1.7	0.274
**b. Longitudinal change (≥ 1 follow‐up; *n* = 187)**				
pTau_181_	ln (estradiol) × time	−0.28 (−0.63, 0.07)	0.18	0.116
	ln (estradiol) × *APOEε4* × time	−0.56 (−1.19, 0.07)	0.32	0.079
	ln (estradiol) × Aβ+ × time	−0.38 (−0.79, 0.03)	0.21	0.07
	ln (estrone) × time	−0.54 (−1.00, −0.08)	0.23	0.021*
	ln (estrone) × *APOEε4* × time	−0.35 (−1.16, 0.45)	0.41	0.388
	ln (estrone) × Aβ+ × time	−0.49 (−1.08, 0.10)	0.3	0.106

*Notes*: (a) Separate single‐hormone models; each biomarker and each interaction term modeled separately. Linear regression models adjusted for baseline age (years), BMI, waist circumference, current hormone replacement therapy use, *APOEε4* carrier status, and Aβ+ (amyloid positivity status); interaction rows represent the additional difference in the hormone association for *APOEε4* carriers versus non‐carriers or for Aβ+ versus Aβ‐, respectively. Robust (Huber–White) standard errors were used. *B* indicates the unstandardized regression coefficient. ln () indicates natural log‐transformed values. * *p *< 0.05; ** *p *< 0.01; *** *p *< 0.001.

(b) Separate single‐hormone models; each biomarker and each interaction term modeled separately. Longitudinal mixed‐effects models were restricted to participants with at least one follow‐up visit and excluded Visit 4 (Year 3; *n* = 3). Age at visit was used as the time metric and mean‐centered; therefore, time terms represent associations with change in pTau_181_ over follow‐up. Models were adjusted for baseline age, BMI, waist circumference, current hormone replacement therapy use, *APOEε4* carrier status, and Aβ+; interaction terms represent differences in hormone‐slope associations by *APOEε4* carrier or Aβ+ status. *B* indicates the unstandardized regression coefficient. ln () indicates natural log‐transformed values. * *p *< 0.05; ** *p *< 0.01; *** *p *< 0.001.

Abbreviations: Aβ, amyloid‐beta; *APOEε4*, apolipoprotein E *ε4*; BMI, body mass index; CI, confidence interval; CSF, cerebrospinal fluid; pTau181, phosphorylated tau 181; SE, standard error.

### 3.3 Estradiol / Estrone and tTau

In baseline cross‐sectional analyses (Table 4a; covariate estimates in Table S4a), higher ln‐transformed estradiol was associated with lower baseline CSF tTau with borderline statistical significance (*β* = −7.027, *p *= 0.050). Baseline estradiol‐tTau associations were stronger among *APOEε4* carriers than non‐carriers (*β* = −15.954, *p* = 0.038; Figure 2B), while there was no evidence of modification by amyloid positivity. Baseline estrone was not associated with baseline tTau. The estrone × *APOEε4* interaction was significant (*β* = −21.390, *p *= 0.047; Figure 2B), whereas modification by amyloid positivity was not supported. In longitudinal models restricted to participants with follow‐up (Table 4b; covariate estimates in Table S4b), neither baseline estradiol nor baseline estrone was associated with overall change in tTau over time. However, amyloid positivity modified longitudinal tTau change in relation to both hormones, with stronger decreases in tTau over follow‐up among amyloid‐positive participants for estradiol (*β* = −4.60, *p* = 0.026) and estrone (*β* = −6.87, *p* = 0.025). *APOEε4* did not modify hormone‐tTau slope associations.

**TABLE 4:** Baseline cross‐sectional and longitudinal associations of serum estradiol and estrone with CSF tTau.

Outcome	Primary exposures	*B* (95% CI)	SE	*p‐*value
**a. Baseline cross‐sectional (Visit 1; *n* = 854)**				
tTau (baseline)	ln (estradiol)	−7.027 (−14.065, 0.011)	3.586	0.05
	ln (estradiol) × *APOEε4*	−15.954 (−31.024, −0.884)	7.678	0.038*
	ln (estradiol) × Aβ+	−9.79 (−25.06, 5.49)	7.78	0.209
	ln (estrone)	−0.400 (−11.086, 10.286)	5.444	0.942
	ln (estrone) × *APOEε4*	−21.390 (−42.448, −0.331)	10.73	0.047*
	ln (estrone) × Aβ+	−12.45 (−42.13, 17.22)	15.12	0.41
**b. Longitudinal change (≥ 1 follow‐up; *n* = 187)**				
tTau	ln (estradiol) × time	−2.02 (−4.62, 0.58)	1.32	0.127
	ln (estradiol) × *APOEε4* × time	−1.34 (−5.79, 3.11)	2.27	0.555
	ln (estradiol) × Aβ+ × time	−4.60 (−8.64, −0.56)	2.06	0.026*
	ln (estrone) × time	−2.87 (−6.58, 0.85)	1.89	0.13
	ln (estrone) × *APOEε4* × time	−5.19 (−11.81, 1.43)	3.38	0.124
	ln (estrone) × Aβ+ × time	−6.87 (−12.89, −0.85)	3.07	0.025*

*Notes*: (a) Separate single‐hormone models; each biomarker and each interaction term modeled separately. Linear regression models adjusted for baseline age (years), BMI, waist circumference, current hormone replacement therapy use, *APOEε4* carrier status, and Aβ+ (amyloid positivity status); interaction rows represent the additional difference in the hormone association for *APOEε4* carriers versus non‐carriers or for Aβ+ versus Aβ‐, respectively. Robust (Huber–White) standard errors were used. *B* indicates the unstandardized regression coefficient. ln () indicates natural log‐transformed values.

* *p *< 0.05; **p < 0.01; ***p < 0.001.

(b) Separate single‐hormone models; each biomarker and each interaction term modeled separately. Longitudinal mixed‐effects models were restricted to participants with at least one follow‐up visit and excluded Visit 4 (Year 3; *n* = 3). Age at visit was used as the time metric and mean‐centered; therefore, time terms represent associations with change in tTau over follow‐up. Models were adjusted for baseline age, BMI, waist circumference, current hormone replacement therapy use, *APOEε4* carrier status, and Aβ+ (amyloid positivity status); interaction terms represent differences in hormone‐slope associations by *APOEε4* carrier or Aβ+ status. ln () indicates natural log‐transformed values. *B* indicates the unstandardized regression coefficient.

*p < 0.05; **p < 0.01; ***p < 0.001.

Abbreviations: Aβ, amyloid‐beta; *APOEε4*, apolipoprotein E *ε4*; BMI, body mass index; CI, confidence interval; CSF, cerebrospinal fluid; SE, standard error; tTau, total Tau.

### 3.4 Sensitivity analyses (excluding current HRT users)

In sensitivity analyses excluding participants who reported current HRT use at baseline (Tables S5–S7), findings were largely consistent with the primary analyses (Tables 2, 3, 4). Associations with Aβ_42_ remained null. Neither estradiol nor estrone was associated with baseline Aβ_42_ (Table 2a vs Table S5a) or with its change over time (Table 2b vs Table S5b), and there was no evidence of effect modification by *APOEε4*.

Associations with pTau_181_ were also largely unchanged. Higher baseline estradiol remained inversely associated with baseline pTau_181_ (Table S6a: *β* = −0.81, *p* = 0.041), and this association was stronger among *APOEε4* carriers (Table S6a: *β* = −1.77, *p* = 0.043). Baseline estradiol was not associated with the overall rate of change in pTau_181_ (Table S6b). However, the estradiol × Aβ+ × time interaction (Table 3b: *β* = −0.38, *p* = 0.070) reached statistical significance in the restricted sample (Table S6b: *β* = −0.49, *p* = 0.027). For estrone, the longitudinal association with pTau_181_ change remained significant (Table S6b: *β* = −0.88, *p* = 0.019), whereas the estrone × Aβ+ × time interaction remained non‐significant (Table S6b: *β* = −0.59, *p* = 0.087).

Findings for tTau followed a similar pattern. Baseline estradiol remained inversely associated with baseline tTau (Table S7a: *β* = −8.00, *p *= 0.038), with a stronger association among *APOEε4* carriers (Table S7a: *β* = −17.89, *p* = 0.037). Neither hormone was associated with the overall rate of change in tTau; however, effect modification by amyloid positivity was more pronounced in the restricted sample for both estradiol (Table S7b: *β* = −5.82, *p* = 0.006, vs Table 4b: *β* = ‐4.60, *p* = 0.026) and estrone (Table S7b: *β* = −9.45, *p* = 0.007, vs Table 4b: *β* = −6.87, *p* = 0.025).

## 4 DISCUSSION

Among female participants in the EPAD LCS, baseline estradiol and estrone were not associated with baseline Aβ_42_ concentrations or its change over time, suggesting limited evidence that peripheral estrogen levels relate to amyloid biomarker dynamics in this cohort. In baseline cross‐sectional analyses, higher baseline estradiol was associated with lower baseline tau biomarkers, and this association was stronger among *APOEε4* carriers. In contrast, in longitudinal mixed‐effects models restricted to participants with at least one follow‐up visit, baseline estradiol was not associated with change in tau over time, and there was no evidence of effect modification by *APOEε4* carrier status. Notably, the longitudinal estradiol–tau association observed when baseline‐only and follow‐up participants were included in the same mixed‐effects models^34^ was attenuated and no longer statistically significant after cross‐sectional and longitudinal analyses were separated, longitudinal models were restricted to participants with follow‐up, and BMI and waist circumference were included as covariates. Accordingly, the more robust finding was the cross‐sectional association of higher baseline estradiol with lower baseline tau, particularly among *APOEε4* carriers, whereas no longitudinal estradiol‐tau association was supported. Because substantial and informative attrition limits longitudinal inference about tau change, the modest association between estrone and slower pTau_181_ increase over follow‐up should be interpreted cautiously. Amyloid positivity did not moderate cross‐sectional hormone‐tau associations but was associated with steeper increases in tau over time.

The inverse association between baseline serum estradiol and baseline CSF tau biomarkers observed here adds to emerging evidence that circulating estradiol may relate to AD‐relevant outcomes in older women. In a study of 110 patients with AD aged ≥60 years, higher serum estradiol was associated with lower neurocognitive impairment severity, consistent with our findings.^35^ Similarly, higher serum estradiol measured by isotope dilution LC‐MS/MS was associated with better Digit Symbol Substitution Test performance among 731 women aged ≥60 years in the National Health and Nutrition Examination Survey.^36^

Biological evidence supports a possible relationship between estradiol signaling and tau‐related pathways, although our cross‐sectional findings do not exclude reverse causation. Estradiol acts through estrogen receptor α (ERα), estrogen receptor β (ERβ), and G protein–coupled estrogen receptor (GPER) in neurons and glia and has been implicated in synaptic plasticity, mitochondrial function, kinase regulation, autophagy, oxidative stress, and neuroinflammatory signaling, including complement, and *APOE*‐related pathways relevant to tau propagation.^37^, ^38^, ^39^, ^40^, ^41^, ^42^, ^43^, ^44^, ^45^, ^46^ These pathways provide plausible mechanisms but do not establish that circulating estradiol directly affects tau pathology in the brain.

Serum estrogen concentrations may not closely reflect central estrogen exposure. Although earlier work suggested stronger plasma–CSF correspondence, with CSF estradiol potentially tracking the unbound plasma fraction,^47^ recent mass‐spectrometric studies have reported weak or modest correlations,^46^, ^48^ and CSF estradiol may partly reflect local central nervous system (CNS) production.^49^ Consistent with this complexity, Hu et al.^46^ reported no association between CSF estradiol or estrone and CSF tTau, while CSF estrogen measures showed different associations with amyloid, neuroinflammatory, and neuroimaging outcomes. In our data, current HRT use was only modestly correlated with serum estradiol and estrone, and multicollinearity among HRT use and measured serum estrogens was low (Tables S8a and S8b in Supplement), supporting interpretation of HRT status and measured hormone concentrations as related but non‐equivalent indicators of estrogen exposure. Thus, although LC‐MS/MS‐measured serum estradiol and estrone provide scalable, quantitative exposure measures, they may only modestly reflect CNS estrogen exposure. The observed serum estradiol–tau associations should therefore be interpreted as associations with circulating hormones rather than direct evidence of central estrogenic activity. Accordingly, our analyses address whether current circulating estrogens relate to concurrent or short‐term longitudinal CSF tau biomarkers; these findings do not establish a mechanistic effect of estrogen within the CNS and do not address lifetime estrogen exposure, menopausal timing or transition‐related effects, or HRT effects.

Estrone findings were less consistent than those for estradiol. Estrone was not associated with baseline pTau_181_ in the cross‐sectional analysis, but higher baseline estrone was associated with a slower longitudinal increase in pTau_181_. Because postmenopausal estrone partly reflects aromatization in adipose tissue, adiposity is a potential confounder.^50^, ^51^ BMI and waist circumference were strongly correlated in the longitudinal sample (*r* = 0.83), but their variance inflation factors indicated moderate rather than severe collinearity. The estrone × time estimate was similar across models excluding both adiposity measures, including BMI only, including waist circumference only, or including both measures (Table S9 in Supplement). Nevertheless, residual confounding and model uncertainty remain possible. Given the smaller longitudinal sample, attrition, absence of a corresponding cross‐sectional association, and lack of correction across the 12 primary tests, this finding is exploratory and requires independent replication.

The cross‐sectional interaction between estrogen and *APOEε4* carrier status suggests that genetic risk may shape the association between circulating estrogens and tau pathology. In baseline analyses, the inverse association between estradiol and tau was stronger among *APOEε4* carriers for both pTau_181_ and tTau. A parallel carrier‐specific pattern was observed for estrone, with the *APOEε4* interaction reaching statistical significance for tTau and borderline significance for pTau_181_ (*p* = 0.05), even though estrone showed no significant overall associations. In contrast, *APOEε4* did not modify the longitudinal associations between either estrogen or change in tau. Figure 2 therefore is useful for visualizing the cross‐sectional moderation pattern, but it should not be interpreted as evidence of modified longitudinal tau trajectories. These moderation findings should therefore be regarded as cross‐sectional and hypothesis‐generating. Prior work suggests that estradiol‐related sterol pathways may differ by *APOEε4* status, with stronger associations of estradiol with 24(S)‐hydroxycholesterol and 27‐hydroxycholesterol among carriers.^17^ Because 24(S)‐hydroxycholesterol is often interpreted as a marker of brain cholesterol turnover, whereas 27‐hydroxycholesterol can act as a selective estrogen receptor modulator, these findings raise the possibility that *APOEε4* modifies the coupling between estrogen signaling and cholesterol‐derived pathways in postmenopausal women.^17^

In present study, neither baseline estradiol nor estrone was associated with baseline CSF Aβ_42_ or with changes in Aβ_42_ over time. These null findings contrast with preclinical evidence that estrogen signaling can influence amyloidogenic processing and Aβ clearance^37^, ^52^, ^53^, ^54^ and that estrogen deficiency or replacement can alter Aβ burden in experimental models.^55^, ^56^, ^57^ However, our study tested a different exposure context: a single baseline serum hormone measure in female participants aged 50 years and older, rather than experimentally manipulated estrogen exposure or repeated measures around the menopausal transition. Thus, the null Aβ_42_ findings may reflect limited correspondence between peripheral baseline estrogens and amyloid biology in this cohort, particularly given low postmenopausal hormone concentrations and the absence of menopausal‐timing data.

In the present study, amyloid positivity was associated with steeper longitudinal increases in CSF tTau, but not pTau_181_. This pattern is partly consistent with evidence that greater amyloid burden drives faster downstream tau accumulation, a process more pronounced in females.^22^, ^23^, ^24^, ^26^, ^58^ Because these effects were confined to a single biomarker and to the attrition‐prone longitudinal slope, we interpret them as hypothesis‐generating rather than confirmatory.

Strengths include LC‐MS/MS measurement of low‐concentration estrogens and standardized CSF biomarker assessment in a large, well‐characterized EPAD LCS cohort. LC‐MS/MS is less susceptible than immunoassays to cross‐reactivity and analytical interference,^31^, ^59^ supporting comparisons within this cohort.

Several limitations should also be considered. Follow‐up attrition was substantial and appeared to be informative, as participants with longitudinal data had higher baseline estrogen concentrations and lower baseline Aβ_42_ than those with baseline‐only data. Accordingly, inferences about longitudinal change should be interpreted cautiously because selection due to differential follow‐up may have biased slope estimates. In addition, estradiol and estrone were measured only at baseline and may not represent cumulative or contemporaneous estrogen exposure during follow‐up. This single‐time‐point exposure assessment further constrains causal interpretation, particularly for longitudinal slope associations. Residual metabolic or cardiovascular confounding may remain despite adjustment for BMI and waist circumference, and peripheral concentrations may not adequately reflect CNS exposure.^46^, ^60^, ^61^ Most participants were likely peri‐ or postmenopausal, but non‐standardized blood collection timing may have introduced additional variability. Although LC‐MS/MS showed favorable internal precision, sensitivity, and recovery, QC materials were prepared in charcoal‐stripped fetal bovine serum, and the assay was not independently compared with a reference procedure using commutable human serum samples. Absolute concentrations therefore may not be comparable with CDC‐standardized procedures or other studies, although use of the same procedure supports internal comparability. HRT was captured only as a binary baseline indicator, and its low prevalence limited evaluation of formulation‐, dose‐, route‐, duration‐, and HRT‐related effects. Finally, the 12 primary cross‐sectional and longitudinal tests were not corrected for multiple comparisons; all findings therefore should be considered exploratory and hypothesis‐generating rather than confirmatory.

Future work should integrate peripheral and CSF hormone measurements with comprehensive AD biomarkers to clarify whether circulating estradiol reflects central estrogen activity or whether peripheral and central compartments exert independent effects and should use repeated hormone assessments over time to link within‐person hormonal change to biomarker trajectories. Integrating molecular markers of estrogen receptor signaling, lipid metabolism, and neuroinflammation may help clarify the pathways through which circulating estrogens relate to tau‐related vulnerability in aging women.

## CONFLICT OF INTEREST STATEMENT

The authors have declared that there are no conflicts of interest in relation to the subject of this study. Author disclosures are available in the Supporting Information.

## CONSENT STATEMENT

All participants in each dataset provided informed consent to participate.

## Supporting information




Supporting Information

Supporting Information
