# Supplementary material for: Associations between serum estradiol and estrone and Alzheimer's disease biomarkers: An analysis in female participants from the European Prevention of Alzheimer's Dementia Longitudinal Cohort Study (EPAD LCS)
Source: Alzheimers Dement. 2026 Sep 13;22(9):e71826. doi: 10.1002/alz.71826 (PMC13572576; doi:10.1002/alz.71826)
Supplement: Supplementary file 1 [file ALZ-22-e71826-s001.pdf]

Supplement

Additional Figures and Tables

Contents

Figure S1. Distribution of serum estradiol and estrone concentrations before and after natural log (ln) transformation 2

Table S1. Skewness and kurtosis of serum estradiol and estrone concentrations before and after natural log (ln) transformation .. 4

Table S2. Baseline cross-sectional and longitudinal associations of serum estradiol and estrone with CSF $A\beta_{42}$ (including covariate estimates).. 5

Table S3. Baseline cross-sectional and longitudinal associations of serum estradiol and estrone with CSF $pTau_{181}$ (including covariate estimates).. 8

Table S4. Baseline cross-sectional and longitudinal associations of serum estradiol and estrone with CSF tTau (including covariate estimates).. 13

Table S5. Baseline cross-sectional and longitudinal associations of serum estradiol and estrone with CSF $A\beta_{42}$ among participants not currently using hormone replacement therapy (HRT) 15

Table S6. Baseline cross-sectional and longitudinal associations of serum estradiol and estrone with CSF $pTau_{181}$ among participants not currently using hormone replacement therapy (HRT) 16

Table S7. Baseline cross-sectional and longitudinal associations of serum estradiol and estrone with CSF tTau among participants not currently using hormone replacement therapy (HRT) 17

Tables S8. Correlations and multicollinearity diagnostics for current hormone replacement therapy (HRT) use and log-transformed estradiol and estrone 18

Table S9. Sensitivity analyses of the longitudinal association between serum estrone and CSF $pTau_{181}$ using alternative adiposity adjustments 19

Figure S1. Distribution of serum estradiol and estrone concentrations before and after natural log (ln) transformation

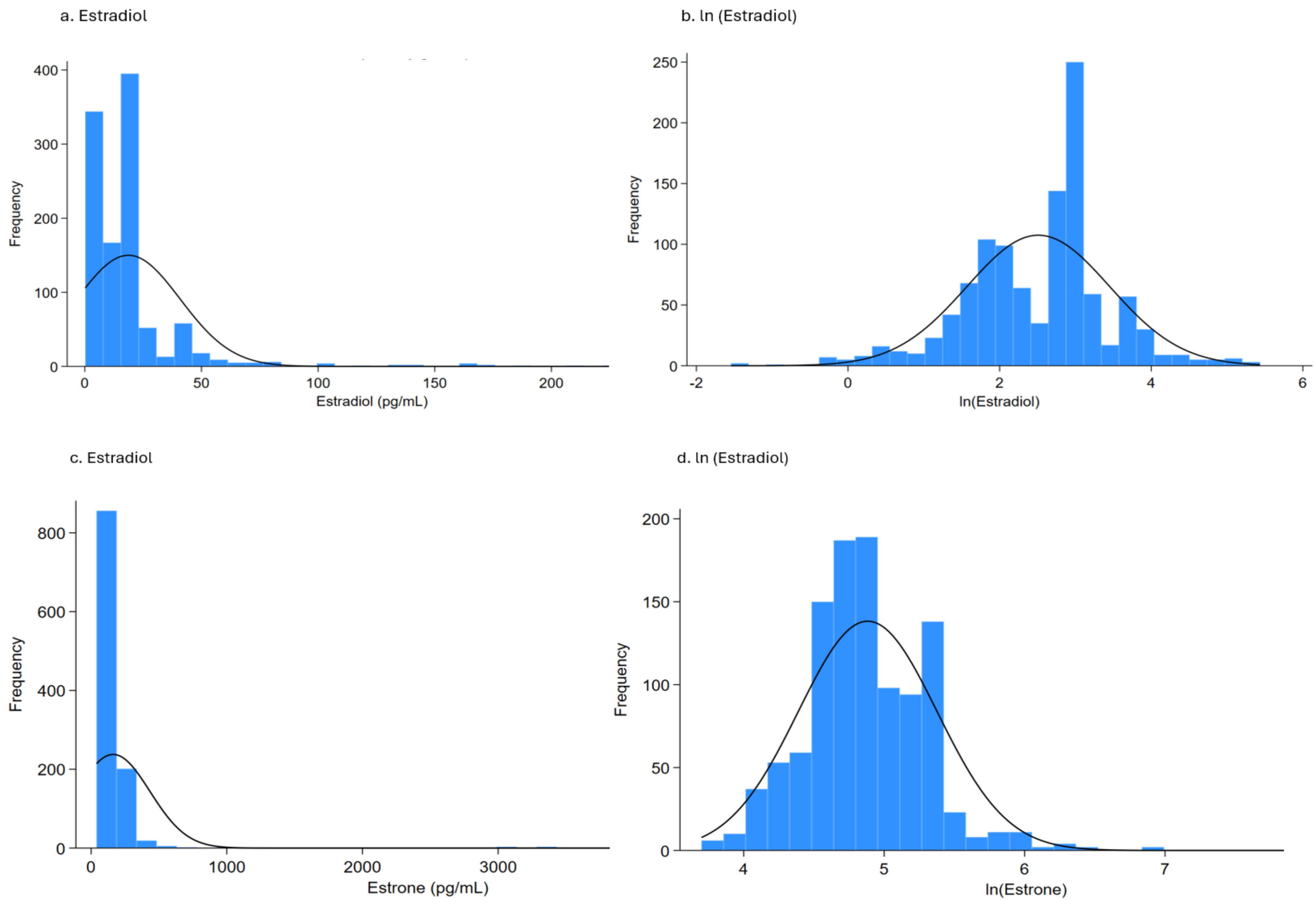

Note. Histograms display the distribution of serum estradiol and estrone concentrations in the analytic sample before (raw values) and after natural log (ln) transformation: (a) histogram of estradiol before ln transformation; (b) histogram of estradiol after ln transformation; (c) histogram of estrone before ln transformation; and (d) histogram of estrone after ln transformation.

Table S1. Skewness and kurtosis of serum estradiol and estrone concentrations before and after natural log (ln) transformation

Descriptive statistic	Estradiol	ln (Estradiol)	Estrone	ln (Estrone)
Skewness	4.57	-0.405	11.4	2.02
SE of skewness	0.0741	0.0741	0.0741	0.0741
Kurtosis	28.6	1.1	142	11.1
SE of kurtosis	0.148	0.148	0.148	0.148

Table S2. Baseline cross-sectional and longitudinal associations of serum estradiol and estrone with CSF Aβ_{42} (including covariate estimates)

a. Baseline cross-sectional (Visit 1; n = 854)

Predictor	**Model 1**			**Model 2**		
	B (95% CI)	SE	p	*B* (95% CI)	SE	p
**Estradiol**						
Intercept	1959.75 (1373.65, 2545.85)	298.6	<.001***	1959.18 (1361.35, 2557.01)	304.6	<.001***
Baseline age (years)	-6.95 (-13.84, -0.07)	3.51	.048*	-6.95 (-13.84, -0.06)	3.51	.048*
BMI	-9.12 (-21.96, 3.71)	6.54	0.163	-9.12 (-21.95, 3.70)	6.53	0.163
Waist circumference	4.36 (-0.52, 9.24)	2.49	0.08	4.36 (-0.52, 9.23)	2.48	0.08
APOEε4 carrier (vs non-carrier)	-377.83 (-474.50, -281.16)	49.25	<.001***	-376.37 (-658.17, -94.56)	143.6	.009**
Current HRT use (vs no)	99.46 (-73.34, 272.27)	88.04	0.259	99.55 (-74.17, 273.27)	88.51	0.261
ln (Estradiol)	-33.60 (-86.37, 19.16)	26.88	0.212	-33.35 (-106.35, 39.64)	37.19	0.37
ln (Estradiol) × *APOEε4* carrier	—	—	—	-0.61 (-101.67, 100.44)	51.49	0.991
**Estrone**						
Intercept	1732.89 (950.39, 2515.40)	398.7	<.001***	1736.45 (854.48, 2618.42)	449.4	<.001***
Baseline age (years)	-6.42 (-13.29, 0.45)	3.5	0.067	-6.42 (-13.30, 0.47)	3.51	0.068
BMI	-10.80 (-23.93, 2.33)	6.69	0.107	-10.80 (-23.96, 2.36)	6.7	0.108
Waist circumference	4.57 (-0.37, 9.52)	2.52	0.07	4.57 (-0.38, 9.52)	2.52	0.07
APOEε4 carrier (vs non-carrier)	-375.41 (-472.37, -278.45)	49.4	<.001***	-384.09 (-1327.62, 559.43)	480.7	0.425
Current HRT use (vs no)	54.04 (-128.90, 236.97)	93.2	0.562	53.96 (-129.54, 237.45)	93.49	0.564
ln (Estrone)	28.35 (-74.08, 130.78)	52.19	0.587	27.59 (-108.09, 163.27)	69.13	0.69
ln (Estrone) × *APOEε4* carrier	—	—	—	1.79 (-187.91, 191.49)	96.65	0.985

Note. Separate single-hormone models; each biomarker and each interaction term modeled separately. Linear regression models adjusted for baseline age (years), body mass index (BMI), waist circumference, current hormone replacement therapy use, and *APOEε4* carrier status; interaction rows represent the additional difference in the hormone association for *APOEε4* carriers versus non-carriers, respectively.

Robust (Huber-White) standard errors were used. *B* indicates the unstandardized regression coefficient. ln () indicates natural log–transformed values. * p< .05; ** p< .01; *** p< .001

b. Longitudinal change (≥ 1 follow-up; n = 187)

Predictor	**Model 1**			**Model 2**		
	B (95% CI)	SE	p	*B* (95% CI)	SE	p
**Estradiol**						
Intercept	4741.16 (2557.03, 6925.29)	1114	<.001***	4788.82 (2591.41, 6986.24)	1121	<.001***
Time	142.01 (-43.83, 327.84)	94.82	0.134	136.56 (-58.26, 331.39)	99.4	0.169
Baseline age (years)	-61.17 (-91.70, -30.64)	15.58	<.001***	-60.70 (-91.30, -30.11)	15.61	<.001***
Baseline age × Time	-1.52 (-3.53, 0.48)	1.02	0.137	-1.53 (-3.55, 0.49)	1.03	0.139
BMI	8.95 (-28.87, 46.77)	19.3	0.643	8.75 (-29.18, 46.67)	19.35	0.651
BMI × Time	-2.13 (-7.62, 3.37)	2.81	0.449	-1.99 (-7.52, 3.53)	2.82	0.479
Waist circumference	4.92 (-9.81, 19.66)	7.52	0.512	5.11 (-9.64, 19.86)	7.53	0.497
Waist × Time	0.73 (-1.53, 2.98)	1.15	0.527	0.68 (-1.59, 2.94)	1.16	0.559
APOEε4 carrier (vs non-carrier)	-321.18 (-506.84, -135.52)	94.73	.001**	-544.80 (-1531.03, 441.43)	503.2	0.279
APOEε4 carrier × Time	-11.10 (-37.79, 15.59)	13.62	0.415	2.30 (-110.15, 114.76)	57.38	0.968
Current HRT use (vs no)	-40.12 (-399.15, 318.90)	183.2	0.827	-44.48 (-403.86, 314.89)	183.4	0.808
Current HRT use × Time	13.02 (-38.75, 64.80)	26.42	0.622	14.39 (-37.63, 66.40)	26.54	0.588
ln (Estradiol)	49.93 (-129.14, 229.00)	91.36	0.585	19.92 (-201.15, 240.99)	112.8	0.86
ln (Estradiol)× Time	5.17 (-16.21, 26.55)	10.91	0.636	7.19 (-21.51, 35.89)	14.64	0.623
ln (Estradiol) × *APOEε4* carrier	—	—	—	75.40 (-257.46, 408.26)	169.8	0.657
ln (Estradiol) × *APOEε4* carrier × Time	—	—	—	-3.92 (-40.69, 32.85)	18.76	0.834
**Estrone**						
Intercept	3272.74 (936.23, 5609.26)	1192	.006**	3336.34 (867.04, 5805.65)	1260	.008**
Time	201.10 (-7.89, 410.10)	106.6	0.059	188.22 (-49.91, 426.35)	121.5	0.121
Baseline age (years)	-59.31 (-89.52, -29.10)	15.41	<.001***	-59.19 (-89.43, -28.94)	15.43	<.001***
Baseline age × Time	-2.17 (-4.04, -0.30)	0.95	.023*	-2.14 (-4.03, -0.26)	0.96	.026*
BMI	5.73 (-31.14, 42.61)	18.81	0.761	6.01 (-30.92, 42.94)	18.84	0.75

BMI × Time	-1.25 (-6.69, 4.18)	2.77	0.651	-1.29 (-6.75, 4.18)	2.79	0.645
Waist circumference	3.65 (-10.99, 18.28)	7.47	0.625	3.61 (-11.04, 18.26)	7.47	0.629
Waist × Time	0.04 (-2.22, 2.29)	1.15	0.976	0.03 (-2.24, 2.30)	1.16	0.978
APOEε4 carrier (vs non-carrier)	-311.03 (-493.70, -128.36)	93.2	.001**	-402.33 (-2674.42, 1869.76)	1159	0.729
APOEε4 carrier × Time	-6.19 (-32.56, 20.18)	13.45	0.645	15.29 (-246.98, 277.56)	133.8	0.909
Current HRT use (vs no)	-281.48 (-668.68, 105.72)	197.6	0.154	-282.71 (-671.06, 105.64)	198.1	0.154
Current HRT use × Time	13.68 (-42.98, 70.33)	28.91	0.636	14.66 (-43.04, 72.36)	29.44	0.619
ln (Estrone)	347.86 (97.85, 597.86)	127.6	.006**	332.49 (17.22, 647.76)	160.9	.039*
ln (Estrone)× Time	6.74 (-21.72, 35.20)	14.52	0.643	9.14 (-26.51, 44.79)	18.19	0.615
APOEε4 carrier × ln (Estrone)	—	—	—	18.06 (-442.08, 478.19)	234.8	0.939
ln (Estrone) × *APOEε4* carrier × Time	—	—	—	-4.23 (-56.50, 48.03)	26.67	0.874

Note. Separate single-hormone models; each biomarker and each interaction term modeled separately. Longitudinal mixed-effects models were restricted to participants with at least one follow-up visit and excluded Visit 4 (Year 3; n = 3). Age at visit was used as the time metric and mean-centered; therefore, time terms represent associations with change in $A\beta_{42}$ over follow-up. Models were adjusted for baseline age, BMI, waist circumference, current hormone replacement therapy use, and *APOEε4* carrier status; interaction terms represent differences in hormone-slope associations by *APOEε4* carrier status. *B* indicates the unstandardized regression coefficient. ln () indicates natural log-transformed values. * p< .05; ** p< .01; *** p< .001

Table S3. Baseline cross-sectional and longitudinal associations of serum estradiol and estrone with CSF $pTau_{181}$ (including covariate estimates)

a. Baseline cross-sectional (Visit 1; n = 854)

Predictor	Model 1			Model 2			Model 3		
	B (95% CI)	SE	p	*B* (95% CI)	SE	p	*B* (95% CI)	SE	p
**Estradiol**									
Intercept	-6.28 (-12.91, 0.35)	3.38	0.064	-7.74 (-14.37, -1.11)	3.38	.022*	-6.79 (-13.58, 0.01)	3.46	0.05
Baseline age (years)	0.46 (0.37, 0.55)	0.04	<.001***	0.46 (0.37, 0.55)	0.04	<.001***	0.46 (0.37, 0.55)	0.05	<.001***
BMI	-0.19 (-0.36, -0.02)	0.09	.027*	-0.19 (-0.36, -0.02)	0.09	.027*	-0.19 (-0.36, -0.02)	0.09	.031*
Waist circumference	0.01 (-0.05, 0.06)	0.03	0.809	0.01 (-0.05, 0.06)	0.03	0.846	0.00 (-0.05, 0.06)	0.03	0.865
APOEε4 carrier (vs non-carrier)	3.43 (1.99, 4.88)	0.74	<.001***	7.18 (2.84, 11.52)	2.21	.001**	3.41 (1.95, 4.86)	0.74	<.001***
Current HRT use (vs no)	0.75 (-1.11, 2.61)	0.95	0.431	0.96 (-0.90, 2.82)	0.95	0.31	0.80 (-1.06, 2.67)	0.95	0.398
Amyloid-positive (vs amyloid-negative)	0.98 (-0.67, 2.63)	0.84	0.245	0.94 (-0.72, 2.59)	0.84	0.267	3.11 (-1.46, 7.67)	2.33	0.182
Ln (Estradiol)	-0.72 (-1.44, -0.002)	0.37	.049*	-0.09 (-0.84, 0.66)	0.38	0.81	-0.44 (-1.26, 0.37)	0.42	0.285
Ln (Estradiol) × *APOEε4* carrier	—	—	—	-1.56 (-3.10, -0.02)	0.78	.047*	—	—	—
ln (Estradiol) × Aβ+	—	—	—	—	—	—	-0.88 (-2.50, 0.75)	0.83	0.289
**Estrone**									
Intercept	-5.82 (-13.97, 2.32)	4.15	0.161	-10.38 (-19.61, -1.15)	4.7	.028*	-8.15 (-16.51, 0.22)	4.26	0.056

Baseline age (years)	0.47 (0.38, 0.55)	0.05	<.001***	0.46 (0.37, 0.55)	0.05	<.001***	0.46 (0.37, 0.55)	0.05	<.001***
BMI	-0.21 (-0.38, -0.04)	0.09	.016*	-0.21 (-0.38, -0.04)	0.09	.015*	-0.20 (-0.37, -0.03)	0.09	.019*
Waist circumference	0.01 (-0.05, 0.06)	0.03	0.739	0.01 (-0.05, 0.07)	0.03	0.729	0.01 (-0.05, 0.06)	0.03	0.764
APOEε4 carrier (vs non-carrier)	3.49 (2.02, 4.95)	0.75	<.001***	14.60 (3.04, 26.16)	5.89	.013*	3.45 (2.00, 4.91)	0.74	<.001***
Current HRT use (vs no)	0.40 (-1.53, 2.32)	0.98	0.686	0.50 (-1.41, 2.41)	0.97	0.609	0.39 (-1.54, 2.32)	0.98	0.691
Amyloid-positive (vs amyloid-negative)	0.94 (-0.72, 2.60)	0.85	0.269	0.89 (-0.76, 2.54)	0.84	0.291	10.00 (-6.57, 26.57)	8.44	0.237
ln (Estrone)	-0.49 (-1.63, 0.64)	0.58	0.394	0.48 (-0.83, 1.79)	0.67	0.473	0.00 (-1.01, 1.00)	0.51	0.996
ln (Estrone) × *APOEε4* carrier	—	—	—	-2.29 (-4.57, -0.005)	1.16	0.05	—	—	—
ln (Estrone) × Aβ+	—	—	—	—	—	—	-1.87 (-5.21, 1.48)	1.7	0.274

Note. Separate single-hormone models; each biomarker and each interaction term modeled separately. Linear regression models adjusted for baseline age (years), body mass index (BMI), waist circumference, current hormone replacement therapy use, *APOEε4* carrier status, and Aβ+ (amyloid positivity status); interaction rows represent the additional difference in the hormone association for *APOEε4* carriers versus non-carriers or for Aβ+ versus Aβ-, respectively. Robust (Huber-White) standard errors were used. *B* indicates the unstandardized regression coefficient. ln () indicates natural log–transformed values. * p< .05; ** p< .01; *** p< .001

b. Longitudinal change (≥ 1 follow-up; n = 187)

Predictor	**Model 1**			**Model 2**			**Model 3**		
	B (95% CI)	SE	p	*B* (95% CI)	SE	p	*B* (95% CI)	SE	p
**Estradiol**									

Intercept	17.09 (-8.03, 42.20)	12.81	0.182	17.00 (-8.21, 42.22)	12.87	0.186	16.71 (-8.74, 42.16)	12.98	0.198
Time	0.30 (-2.53, 3.12)	1.44	0.837	-0.43 (-3.38, 2.51)	1.5	0.773	0.02 (-2.78, 2.82)	1.43	0.991
Baseline age (years)	0.02 (-0.31, 0.34)	0.17	0.921	0.04 (-0.29, 0.36)	0.17	0.824	-0.01 (-0.34, 0.31)	0.17	0.943
Baseline age × Time	0.00 (-0.03, 0.03)	0.02	0.91	0.00 (-0.03, 0.03)	0.02	0.94	0.00 (-0.03, 0.03)	0.02	0.825
BMI	0.04 (-0.53, 0.61)	0.29	0.888	0.00 (-0.57, 0.57)	0.29	0.992	-0.01 (-0.58, 0.56)	0.29	0.975
BMI × Time	-0.04 (-0.12, 0.04)	0.04	0.339	-0.04 (-0.12, 0.04)	0.04	0.353	-0.05 (-0.13, 0.03)	0.04	0.258
Waist circumference	0.03 (-0.19, 0.25)	0.11	0.791	0.04 (-0.18, 0.26)	0.11	0.723	0.04 (-0.18, 0.26)	0.11	0.716
Waist × Time	0.02 (-0.01, 0.05)	0.02	0.233	0.02 (-0.02, 0.05)	0.02	0.286	0.02 (-0.01, 0.05)	0.02	0.188
APOEε4 carrier (vs non-carrier)	1.41 (-1.41, 4.22)	1.44	0.328	-1.30 (-16.57, 13.97)	7.79	0.867	1.56 (-1.24, 4.37)	1.43	0.274
APOEε4 carrier × Time	0.70 (0.30, 1.10)	0.2	.001**	2.37 (0.50, 4.25)	0.96	.013*	0.73 (0.34, 1.12)	0.2	<.001***
Current HRT use (vs no)	-0.25 (-5.65, 5.15)	2.75	0.927	-0.37 (-5.77, 5.02)	2.75	0.892	-0.70 (-6.12, 4.71)	2.76	0.799
Current HRT use × Time	0.55 (-0.21, 1.31)	0.39	0.157	0.58 (-0.19, 1.35)	0.39	0.141	0.44 (-0.32, 1.20)	0.39	0.255
Amyloid-positive (vs amyloid-negative)	0.32 (-0.97, 1.61)	0.66	0.631	0.33 (-0.95, 1.61)	0.65	0.618	8.21 (-4.89, 21.32)	6.69	0.219
Amyloid-positive × Time	0.07 (-0.13, 0.28)	0.1	0.465	0.08 (-0.12, 0.28)	0.1	0.433	1.25 (-0.05, 2.55)	0.66	0.059
ln (Estradiol)	-1.20 (-3.93, 1.53)	1.39	0.39	-1.53 (-4.88, 1.82)	1.71	0.371	-0.32 (-3.52, 2.88)	1.63	0.846

ln (Estradiol)× Time	-0.28 (-0.63, 0.07)	0.18	0.116	0.00 (-0.47, 0.47)	0.24	0.996	-0.14 (-0.52, 0.23)	0.19	0.449
ln (Estradiol) × *APOEε4* carrier	—	—	—	0.79 (-4.41, 5.98)	2.65	0.767	—	—	—
ln (Estradiol) × *APOEε4* carrier × Time	—	—	—	-0.56 (-1.19, 0.07)	0.32	0.079	—	—	—
ln (Estradiol) × Aβ+	—	—	—	—	—	—	-2.79 (-7.29, 1.71)	2.29	0.224
ln (Estradiol) × Aβ+ × Time	—	—	—	—	—	—	-0.38 (-0.79, 0.03)	0.21	0.07
**Estrone**									
Intercept	15.85 (-12.68, 44.39)	14.56	0.276	7.96 (-22.67, 38.59)	15.63	0.61	13.80 (-15.28, 42.87)	14.83	0.352
Time	1.80 (-1.47, 5.07)	1.67	0.281	1.41 (-2.21, 5.04)	1.85	0.445	1.50 (-1.73, 4.74)	1.65	0.363
Baseline age (years)	0.06 (-0.26, 0.38)	0.16	0.704	0.04 (-0.28, 0.36)	0.16	0.812	0.04 (-0.28, 0.37)	0.16	0.786
Baseline age × Time	0.00 (-0.03, 0.03)	0.02	0.978	0.00 (-0.03, 0.03)	0.02	0.991	-0.00 (-0.03, 0.03)	0.02	0.873
BMI	-0.01 (-0.58, 0.56)	0.29	0.967	-0.01 (-0.58, 0.55)	0.29	0.961	-0.02 (-0.59, 0.55)	0.29	0.944
BMI × Time	-0.04 (-0.12, 0.05)	0.04	0.388	-0.03 (-0.11, 0.05)	0.04	0.436	-0.04 (-0.12, 0.04)	0.04	0.366
Waist circumference	0.06 (-0.16, 0.28)	0.11	0.578	0.05 (-0.17, 0.27)	0.11	0.649	0.06 (-0.16, 0.28)	0.11	0.593
Waist × Time	0.02 (-0.01, 0.05)	0.02	0.21	0.02 (-0.01, 0.05)	0.02	0.272	0.02 (-0.01, 0.05)	0.02	0.216
APOEε4 carrier (vs non-carrier)	1.00 (-1.83, 3.83)	1.44	0.49	25.71 (-8.23, 59.65)	17.32	0.138	1.02 (-1.81, 3.84)	1.44	0.48

APOEε4 carrier × Time	0.67 (0.27, 1.07)	0.2	.001**	2.42 (-1.60, 6.44)	2.05	0.239	0.68 (0.29, 1.07)	0.2	.001**
Current HRT use (vs no)	-0.00 (-5.90, 5.90)	3.01	0.999	-0.36 (-6.24, 5.53)	3	0.905	-0.18 (-6.07, 5.71)	3.01	0.952
Current HRT use × Time	0.87 (0.03, 1.71)	0.43	.041*	0.82 (-0.01, 1.66)	0.43	0.053	0.79 (-0.05, 1.62)	0.42	0.064
Amyloid-positive (vs amyloid-negative)	0.29 (-0.99, 1.58)	0.65	0.653	0.28 (-1.00, 1.56)	0.65	0.669	17.71 (-15.72, 51.15)	17.06	0.299
Amyloid-positive × Time	0.07 (-0.13, 0.27)	0.1	0.479	0.07 (-0.13, 0.27)	0.1	0.476	2.52 (-0.43, 5.48)	1.51	0.094
ln (Estrone)	-1.31 (-5.15, 2.52)	1.96	0.503	0.80 (-4.12, 5.71)	2.51	0.75	-0.55 (-4.59, 3.49)	2.06	0.79
ln (Estrone)× Time	-0.54 (-1.00, -0.08)	0.23	.021*	-0.43 (-1.03, 0.17)	0.3	0.158	-0.43 (-0.90, 0.04)	0.24	0.076
ln (Estrone) × *APOEε4* carrier	—	—	—	-5.01 (-11.88, 1.86)	3.5	0.153	—	—	—
ln (Estrone) × *APOEε4* carrier × Time	—	—	—	-0.35 (-1.16, 0.45)	0.41	0.388	—	—	—
ln (Estrone) × Aβ+	—	—	—	—	—	—	-3.61 (-10.46, 3.24)	3.5	0.301
ln (Estrone) × Aβ+ × Time	—	—	—	—	—	—	-0.49 (-1.08, 0.10)	0.3	0.106

Note. Separate single-hormone models; each biomarker and each interaction term modeled separately. Longitudinal mixed-effects models were restricted to participants with at least one follow-up visit and excluded Visit 4 (Year 3; n = 3). Age at visit was used as the time metric and mean-centered; therefore, time terms represent associations with change in $pTau_{181}$ over follow-up. Models were adjusted for baseline age, BMI, waist circumference, current hormone replacement therapy use, *APOEε4* carrier status, and Aβ+; interaction terms represent differences in hormone–slope associations by *APOEε4* carrier or Aβ+ status. *B* indicates the unstandardized regression coefficient. ln () indicates natural log–transformed values. * p< .05; ** p< .01; *** p< .001

Table S4. Baseline cross-sectional and longitudinal associations of serum estradiol and estrone with CSF tTau (including covariate estimates)

a. Baseline cross-sectional (Visit 1; n = 854)

Predictor	Model 1			Model 2			Model 3		
	B (95% CI)	SE	p	*B* (95% CI)	SE	p	*B* (95% CI)	SE	p
**Estradiol**									
Intercept	-33.80 (-98.20, 30.59)	32.8	0.303	-48.83 (-112.50, 14.84)	32.44	0.133	-39.49 (-105.33, 26.34)	33.54	0.239
Baseline age (years)	4.72 (3.88, 5.57)	0.43	<.001***	4.72 (3.88, 5.56)	0.43	<.001***	4.71 (3.86, 5.56)	0.43	<.001***
BMI	-2.07 (-3.63, -0.51)	0.8	.010*	-2.04 (-3.58, -0.50)	0.78	.010*	-2.02 (-3.59, -0.45)	0.8	.012*
Waist circumference	0.09 (-0.45, 0.63)	0.27	0.746	0.07 (-0.45, 0.60)	0.27	0.783	0.07 (-0.47, 0.60)	0.27	0.809
APOEε4 carrier (vs non-carrier)	33.91 (20.20, 47.61)	6.98	<.001***	72.27 (29.79, 114.76)	21.65	.001**	33.61 (19.84, 47.38)	7.02	<.001***
Current HRT use (vs no)	7.84 (-10.14, 25.82)	9.16	0.392	10.04 (-8.02, 28.09)	9.2	0.276	8.47 (-9.62, 26.55)	9.21	0.358
Amyloid-positive (vs amyloid-negative)	-7.63 (-22.69, 7.42)	7.67	0.32	-8.07 (-23.14, 6.99)	7.68	0.293	16.06 (-27.26, 59.39)	22.07	0.467
ln (Estradiol)	-7.03 (-14.06, 0.01)	3.59	0.05	-0.56 (-7.90, 6.78)	3.74	0.881	-3.91 (-12.24, 4.41)	4.24	0.356
ln (Estradiol) × *APOEε4* carrier	—	—	—	-15.95 (-31.02, -0.88)	7.68	.038*	—	—	—
ln (Estradiol) × Aβ+	—	—	—	—	—	—	-9.79 (-25.06, 5.49)	7.78	0.209
**Estrone**									
Intercept	-50.66 (-130.64, 29.32)	40.8	0.214	-93.30 (-182.34, -4.26)	45.36	.040*	-66.16 (-148.63, 16.30)	42.01	0.116
Baseline age (years)	4.81 (3.95, 5.66)	0.44	<.001***	4.79 (3.94, 5.64)	0.43	<.001***	4.80 (3.94, 5.65)	0.44	<.001***
BMI	-2.31 (-3.87, -0.74)	0.8	.004**	-2.33 (-3.90, -0.76)	0.8	.004**	-2.27 (-3.84, -0.70)	0.8	.005**

Waist circumference	0.12 (-0.41, 0.66)	0.27	0.654	0.13 (-0.41, 0.66)	0.27	0.645	0.12 (-0.42, 0.65)	0.27	0.671
APOEε4 carrier (vs non-carrier)	34.43 (20.58, 48.28)	7.06	<.001***	138.47 (31.46, 245.47)	54.52	.011*	34.21 (20.39, 48.03)	7.04	<.001***
Current HRT use (vs no)	1.94 (-16.64, 20.51)	9.46	0.838	2.88 (-15.57, 21.34)	9.4	0.759	1.90 (-16.76, 20.55)	9.5	0.842
Amyloid-positive (vs amyloid-negative)	-7.96 (-23.10, 7.17)	7.71	0.302	-8.38 (-23.49, 6.72)	7.7	0.276	52.47 (-94.64, 199.58)	74.95	0.484
ln (Estrone)	-0.40 (-11.09, 10.29)	5.44	0.942	8.71 (-4.12, 21.54)	6.54	0.183	2.87 (-7.44, 13.19)	5.25	0.585
ln (Estrone) × *APOEε4* carrier	—	—	—	-21.39 (-42.45, -0.33)	10.73	.047*	—	—	—
ln (Estrone) × Aβ+	—	—	—	—	—	—	-12.45 (-42.13, 17.22)	15.12	0.41

Note. Separate single-hormone models; each biomarker and each interaction term modeled separately. Linear regression models adjusted for baseline age (years), body mass index (BMI), waist circumference, current hormone replacement therapy use, *APOEε4* carrier status, and Aβ+ (amyloid positivity status); interaction rows represent the additional difference in the hormone association for *APOEε4* carriers versus non-carriers or for Aβ+ versus Aβ-, respectively. Robust (Huber-White) standard errors were used. *B* indicates the unstandardized regression coefficient. ln () indicates natural log–transformed values. * p< .05; ** p< .01; *** p< .001

Table S5. Baseline cross-sectional and longitudinal associations of serum estradiol and estrone with CSF $A\beta_{42}$ among participants not currently using hormone replacement therapy (HRT)

a. Baseline cross-sectional analysis excluding current HRT users (Visit 1; n =791)

Outcome	Primary exposures	*B* (95% CI)	SE	*p*
$A\beta_{42}$ (baseline)	ln (Estradiol)	-32.84 (-88.69, 23.02)	28.5	0.25
	ln (Estradiol) × *APOEε4*	16.84 (-93.24, 126.93)	56.1	0.76
	ln (Estrone)	18.15 (-106.99, 143.29)	63.8	0.78
	ln (Estrone) × *APOEε4*	-16.06 (-271.05, 238.93)	130	0.9

Note. Separate single-hormone models; each biomarker and each interaction term modeled separately. Linear regression models adjusted for baseline age (years), body mass index (BMI), waist circumference, and *APOEε4* carrier status; interaction rows represent the additional difference in the hormone association for *APOEε4* carriers versus non-carriers, respectively. Robust (Huber-White) standard errors were used. *B* indicates the unstandardized regression coefficient. * $p< .05$; ** $p< .01$; *** $p< .001$

b. Longitudinal change excluding current HRT users (≥ 1 follow-up; n =172)

Outcome	Primary exposures	*B* (95% CI)	SE	*p*
$A\beta_{42}$	ln (Estradiol) × Time	3.40 (-19.48, 26.29)	11.7	0.77
	ln (Estradiol) × *APOEε4* × Time	14.88 (-25.29, 55.06)	20.5	0.47
	ln (Estrone) × Time	20.96 (-19.66, 61.58)	20.7	0.31
	ln (Estrone) × *APOEε4* × Time	-2.58 (-80.10, 74.94)	39.6	0.95

Note. Separate single-hormone models; each biomarker and each interaction term modeled separately. Longitudinal mixed-effects models were restricted to participants with at least one follow-up visit, excluding Visit 4 and current HRT users. Age at visit was used as the time metric and mean-centered; therefore, time terms represent associations with change in $A\beta_{42}$ over follow-up. Models were adjusted for baseline age, BMI, waist circumference, and *APOEε4* carrier status; interaction terms represent differences in hormone–slope associations by *APOEε4* carrier status. *B* indicates the unstandardized regression coefficient. * $p< .05$; ** $p< .01$; *** $p< .001$

Table S6. Baseline cross-sectional and longitudinal associations of serum estradiol and estrone with CSF $pTau_{181}$ among participants not currently using hormone replacement therapy (HRT)

a. Baseline cross-sectional analysis excluding current HRT users (Visit 1; n = 791)

Outcome	Primary exposures	*B* (95% CI)	SE	*p*
$pTau_{181}$ (baseline)	ln (Estradiol)	-0.81 (-1.58, -0.03)	0.39	0.041*
	ln (Estradiol) × *APOEε4*	-1.77 (-3.47, -0.06)	0.87	0.043*
	ln (Estradiol) × Aβ+	-1.17 (-2.93, 0.60)	0.9	0.194
	ln (Estrone)	-0.46 (-1.95, 1.03)	0.76	0.542
	ln (Estrone) × *APOEε4*	-3.16 (-6.39, 0.07)	1.65	0.055
	ln (Estrone) × Aβ+	-2.65 (-6.38, 1.07)	1.9	0.162

Note. Separate single-hormone models; each biomarker and each interaction term modeled separately. Linear regression models adjusted for baseline age (years), body mass index (BMI), waist circumference, *APOEε4* carrier status, and Aβ+ (amyloid positivity status); interaction rows represent the additional difference in the hormone association for *APOEε4* carriers versus non-carriers or for Aβ+ versus Aβ-, respectively. Robust (Huber-White) standard errors were used. *B* indicates the unstandardized regression coefficient. * $p< .05$; ** $p< .01$; *** $p< .001$

b. Longitudinal change excluding current HRT users (≥ 1 follow-up; n = 172)

Outcome	Primary exposures	*B* (95% CI)	SE	*p*
$pTau_{181}$	ln (Estradiol) × Time	-0.31 (-0.72, 0.09)	0.21	0.13
	ln (Estradiol) × *APOEε4* × Time	-0.63 (-1.42, 0.15)	0.4	0.115
	ln (Estradiol) × Aβ+ × Time	-0.49 (-0.92, -0.06)	0.22	0.027*
	ln (Estrone) × Time	-0.88 (-1.62, -0.15)	0.38	0.019*
	ln (Estrone) × *APOEε4* × Time	-0.41 (-1.78, 0.96)	0.7	0.56
	ln (Estrone) × Aβ+ × Time	-0.59 (-1.27, 0.09)	0.34	0.087

Note. Separate single-hormone models; each biomarker and each interaction term modeled separately. Longitudinal mixed-effects models were restricted to participants with at least one follow-up visit, excluding Visit 4 and current HRT users. Age at visit was used as the time metric and mean-centered; therefore, time terms represent associations with change in $pTau_{181}$ over follow-up. Models were adjusted for baseline age, BMI, waist circumference, *APOEε4* carrier status, and Aβ+; interaction terms represent differences in hormone–slope associations by *APOEε4* carrier or Aβ+ status. *B* indicates the unstandardized regression coefficient. * $p< .05$; ** $p< .01$; *** $p< .001$

Table S7. Baseline cross-sectional and longitudinal associations of serum estradiol and estrone with CSF tTau among participants not currently using hormone replacement therapy (HRT)

a. Baseline cross-sectional analysis excluding current HRT users (Visit 1; n =791)

Outcome	Primary exposures	*B* (95% CI)	SE	*p*
tTau (baseline)	ln (Estradiol)	-8.00 (-15.56, -0.45)	3.85	0.038*
	ln (Estradiol) × *APOEε4*	-17.89 (-34.72, -1.07)	8.57	0.037*
	ln (Estradiol) × Aβ+	-13.01 (-29.66, 3.63)	8.48	0.125
	ln (Estrone)	0.87 (-12.99, 14.74)	7.06	0.902
	ln (Estrone) × *APOEε4*	-28.15 (-58.02, 1.72)	15.22	0.065
	ln (Estrone) × Aβ+	-21.91 (-55.48, 11.66)	17.1	0.201

Note. Separate single-hormone models; each biomarker and each interaction term modeled separately. Linear regression models adjusted for baseline age (years), body mass index (BMI), waist circumference, *APOEε4* carrier status, and Aβ+ (amyloid positivity status); interaction rows represent the additional difference in the hormone association for *APOEε4* carriers versus non-carriers or for Aβ+ versus Aβ−, respectively. Robust (Huber–White) standard errors were used. *B* indicates the unstandardized regression coefficient. * *p*< .05; ** *p*< .01; *** *p*< .001

b. Longitudinal change excluding current HRT users (≥ 1 follow-up; n = 172)

Outcome	Primary exposures	*B* (95% CI)	SE	*p*
tTau	ln (Estradiol) × Time	-2.93 (-6.52, 0.66)	1.83	0.11
	ln (Estradiol) × *APOEε4* × Time	-3.17 (-10.31, 3.97)	3.64	0.385
	ln (Estradiol) × Aβ+ × Time	-5.82 (-10.00, -1.63)	2.13	0.006**
	ln (Estrone) × Time	-6.28 (-13.05, 0.49)	3.46	0.069
	ln (Estrone) × *APOEε4* × Time	-6.56 (-18.94, 5.83)	6.32	0.299
	ln (Estrone) × Aβ+ × Time	-9.45 (-16.37, -2.53)	3.53	0.007**

Note. Separate single-hormone models; each biomarker and each interaction term modeled separately. Longitudinal mixed-effects models were restricted to participants with at least one follow-up visit, excluding Visit 4 and current HRT users. Age at visit was used as the time metric and mean-centered; therefore, time terms represent associations with change in tTau over follow-up. Models were adjusted for baseline age, BMI, waist circumference, *APOEε4* carrier status, and Aβ+; interaction terms represent differences in hormone–slope associations by *APOEε4* carrier or Aβ+ status. *B* indicates the unstandardized regression coefficient. * *p*< .05; ** *p*< .01; *** *p*< .001

Tables S8. Correlations and multicollinearity diagnostics for current hormone replacement therapy (HRT) use and log-transformed estradiol and estrone

a. Pearson correlations among current HRT use and natural log-transformed estradiol and estrone

	HRT current use	Estradiol (log)	Estrone (log)
HRT current use (yes/no)	1		
Estradiol (ln-transformed, pg/mL)	0.25***	1	
Estrone (ln-transformed, pg/mL)	0.36***	0.54***	1

* p< .05; ** p< .01; *** p< .001

b. Variance inflation factors (VIFs) for current HRT use and natural log-transformed estradiol and estrone

Variable	VIF	1/VIF
HRT current use (yes/no)	1.15	0.87
Estradiol (ln-transformed, pg/mL)	1.43	0.7
Estrone (ln-transformed, pg/mL)	1.54	0.65
Mean VIF	1.37	

Note: variance inflation factors (VIF)

Table S9. Sensitivity analyses of the longitudinal association between serum estrone and CSF $pTau_{181}$ using alternative adiposity adjustments

a. Model excluding both BMI and waist circumference (n = 187)

Fixed effect	*B* (95% CI)	SE	*p*
Baseline age	0.077 (-0.241, 0.394)	0.162	0.637
Time	2.248 (-0.903, 5.399)	1.608	0.162
Baseline age × Time	0.005 (-0.026, 0.035)	0.015	0.764
APOEε4 carrier	0.857 (-1.962, 3.676)	1.438	0.551
APOEε4 carrier × Time	0.661 (0.259, 1.063)	0.205	0.001**
Current HRT use	-0.697 (-6.452, 5.058)	2.936	0.812
Current HRT use × Time	0.780 (-0.043, 1.603)	0.42	0.063
Amyloid-positive	0.331 (-0.947, 1.608)	0.652	0.612
Amyloid-positive × Time	0.065 (-0.135, 0.265)	0.102	0.523
ln (Estrone)	-0.606 (-4.292, 3.080)	1.881	0.747
ln (Estrone) × Time	-0.499 (-0.946, -0.051)	0.228	0.029*
Intercept	16.664 (-11.336, 44.664)	14.29	0.243

Note. Longitudinal mixed-effects model restricted to participants with ≥1 follow-up visit, excluding Visit 4; age at visit was the mean-centered time metric. The model included random intercepts/slopes for time and adjusted for baseline age, current HRT use, *APOEε4* carrier status, amyloid positivity, and ln-transformed estrone (time interactions included for all covariates), excluding BMI and waist circumference. B indicates the unstandardized fixed-effect coefficient. BMI, body mass index; CI, confidence interval; HRT, hormone replacement therapy; $pTau_{181}$, phosphorylated tau 181; SE, standard error. * $p< .05$; ** $p< .01$; *** $p< .001$

b. Model including BMI and excluding waist circumference (n = 187)

Fixed effect	*B* (95% CI)	SE	*p*
Baseline age	0.074 (-0.244, 0.392)	0.162	0.648
Time	2.187 (-1.025, 5.399)	1.639	0.182
Baseline age × Time	0.004 (-0.026, 0.035)	0.015	0.773
BMI	0.112 (-0.213, 0.437)	0.166	0.499
BMI × Time	0.007 (-0.038, 0.053)	0.023	0.749
APOEε4 carrier	0.906 (-1.917, 3.728)	1.44	0.529
APOEε4 carrier × Time	0.659 (0.257, 1.061)	0.205	0.001**
Current HRT use	-0.531 (-6.306, 5.244)	2.946	0.857
Current HRT use × Time	0.786 (-0.045, 1.618)	0.424	0.064
Amyloid-positive	0.360 (-0.920, 1.641)	0.653	0.581
Amyloid-positive × Time	0.065 (-0.134, 0.265)	0.102	0.522
ln (Estrone)	-0.892 (-4.673, 2.889)	1.929	0.644
ln (Estrone) × Time	-0.522 (-0.980, -0.064)	0.234	0.025*

Intercept	15.319 (−12.980, 43.618)	14.44	0.289

Note. Longitudinal mixed-effects model restricted to participants with ≥1 follow-up visit, excluding Visit 4; age at visit was the mean-centered time metric. The model included random intercepts/slopes for time and adjusted for baseline age, BMI, current HRT use, *APOEε4* carrier status, amyloid positivity, and ln-transformed estrone (time interactions included for all covariates), excluding waist circumference. B indicates the unstandardized fixed-effect coefficient. BMI, body mass index; CI, confidence interval; HRT, hormone replacement therapy; $pTau_{181}$, phosphorylated tau 181; SE, standard error. * p< .05; ** p< .01; *** p< .001

c. Model including waist circumference and excluding BMI (n = 187)

**Fixed effect**	***B* (95% CI)**	**SE**	***p***
Baseline age	0.061 (-0.258, 0.379)	0.162	0.71
Time	1.916 (-1.363, 5.194)	1.673	0.252
Baseline age × Time	0.003 (-0.028, 0.033)	0.016	0.868
Waist circumference	0.061 (-0.066, 0.188)	0.065	0.346
Waist circumference × Time	0.009 (-0.009, 0.027)	0.009	0.337
APOEε4 carrier	0.868 (-1.951, 3.687)	1.438	0.546
APOEε4 carrier × Time	0.677 (0.274, 1.080)	0.206	0.001**
Current HRT use	-0.180 (-6.032, 5.671)	2.985	0.952
Current HRT use × Time	0.868 (0.027, 1.710)	0.429	0.043
Amyloid-positive	0.358 (-0.918, 1.634)	0.651	0.583
Amyloid-positive × Time	0.071 (-0.129, 0.270)	0.102	0.487
ln (Estrone)	-1.193 (-5.016, 2.630)	1.951	0.541
ln (Estrone) × Time	-0.565 (-1.026, -0.104)	0.235	0.016*
Intercept	15.247 (-13.246, 43.740)	14.54	0.294

Note. Longitudinal mixed-effects model restricted to participants with ≥1 follow-up visit, excluding Visit 4; age at visit was the mean-centered time metric. The model included random intercepts/slopes for time and adjusted for baseline age, waist circumference, current HRT use, *APOEε4* carrier status, amyloid positivity, and ln-transformed estrone (time interactions included for all covariates); BMI was excluded. B indicates the unstandardized fixed-effect coefficient. BMI, body mass index; CI, confidence interval; HRT, hormone replacement therapy; $pTau_{181}$, phosphorylated tau 181; SE, standard error. * p< .05; ** p< .01; *** p< .001
